# Effect of Atracurium versus Cisatracurium on QT interval changes in patients undergoing cataract surgery: a randomized clinical trial

**DOI:** 10.1186/s12871-024-02820-2

**Published:** 2024-11-27

**Authors:** Mehdi Karimi, Ali Ghaheri, Kianmehr Saleh, Zahra Cheraghi, Afshin Farahanchi

**Affiliations:** 1 Faculty of Medicine, Bogomolets National Medical University (NMU), Kyiv, Ukraine; 2 Faculty of Medicine, Hamadan University of Medical Science (UMSHA), Hamadan, Iran; 3 Department of Epidemiology, Faculty of Health, Hamadan University of Medical Science (UMSHA), Hamadan, Iran; 4 Department of Anesthesiology and Critical Care, Faculty of Medicine, Hamadan University of Medical Science (UMSHA), Hamadan, Iran

**Keywords:** Muscle relaxant, Atracurium, Cisatracurium: anesthesia, ECG, QT interval, Surgery

## Abstract

**Background:**

Muscle relaxants are used during surgery, but their impact on ECG may differ, potentially affecting cardiac safety. This study aimed to compare the effects of Atracurium versus Cisatracurium on QT interval changes in patients undergoing cataract surgery.

**Method:**

This double-blind, parallel-group randomized clinical trial (RCT) was conducted in 2023 in Hamadan, Iran. A total of 80 patients undergoing cataract surgery under general anesthesia were randomly assigned to receive either Atracurium (*n* = 40) or Cisatracurium (*n* = 40). QT interval changes were measured at four time points to assess and compare the corrected QT interval (QTc) between the two groups. Data were analyzed using SPSS version 29, and a *p*-value < 0.05 was deemed significant.

**Results:**

Cisatracurium demonstrated significant reductions in QTc from pre-anesthesia to post-anesthesia and through recovery, with values of -9.325 ms (*P* = 0.045), -9.925 ms (*P* = 0.038), and − 19.359 ms (*P* = 0.016), respectively. Atracurium also showed reductions but a notable increase in QTc after anesthesia to the end of surgery (32.322 ms, *P* = 0.0019). Throughout the procedure, Cisatracurium maintained shorter QTc intervals compared to Atracurium (e.g., T0: 420.07 ms vs. 434.75 ms, *P* = 0.03), but post-recovery, no significant differences were observed (Cisatracurium: 440.05 ms; Atracurium: 439.80 ms, *P* = 0.489).

**Conclusions:**

Atracurium causes more QT prolongation than Cisatracurium. While both affect QTc intervals, Cisatracurium has a more stable impact on cardiac repolarization, making it safer for patients at risk of QT prolongation. Cisatracurium’s minimal impact on cardiovascular function, especially in patients with low ejection fraction, makes it the preferred choice for maintaining cardiac stability.

**Trial registration:**

IRCT20120215009014N441.

## Introduction

The QT interval, measured from the onset of ventricular depolarization to the end of ventricular repolarization, is a key electrocardiographic parameter used to assess the risk of cardiac arrhythmias [1, 2]. QT interval prolongation, especially when corrected for heart rate (QTc), is associated with a heightened risk of developing torsades de pointes (TdP), a potentially life-threatening polymorphic ventricular tachycardia that can lead to sudden cardiac death [3, 4]. During anesthesia, the concern for QTc prolongation becomes particularly important, as patients are exposed to multiple perioperative risk factors, including electrolyte disturbances, surgical stress, and anesthetic agents’ interactions among sedatives, analgesics, and muscle relaxants [4–7].

Recent clinical trials and reviews have highlighted the importance of monitoring QTc during anesthesia, particularly in patients with preexisting risk factors for arrhythmias [8, 9]. For example, patients with congenital long QT syndrome (LQTS) who are undergoing surgery are at heightened risk for adverse cardiac events, especially when exposed to QT-prolonging anesthetic agents [3, 10].

Several classes of medications have been implicated in QTc prolongation [11], such as nondepolarizing neuromuscular blocking agents (NMBAs) of the benzylisoquinolinium class, such as pancuronium, vecuronium, and atracurium, garnering specific attention in the anesthesiology community [12, 13]. Owing to its propensity to cause histamine release, Atracurium can lead to cardiovascular instability, including changes in heart rate and blood pressure, potentially exacerbating QT prolongation [5, 14]. In contrast, cisatracurium, a stereoisomer of atracurium, is often favored for its reduced histamine release and more stable hemodynamic profile, although its exact effects on the QT interval during anesthesia remain unclear [8, 12].

NMBAs such as rocuronium, mivacurium, and Atracurium have been previously shown to increase the QT interval [15]. However, the comparative effects of Atracurium and Cisatracurium on QTc changes during general anesthesia have not been thoroughly studied, leaving a critical gap in perioperative cardiac risk assessment [10, 14].

The literature has demonstrated conflicting results regarding the extent of QT prolongation induced by these agents. Compared with cisatracurium, the hemodynamic effects of Atracurium have been associated with greater QTc prolongation, often described as having a more favorable cardiac safety profile [4–6, 8]. However, these findings are not universally agreed upon, and further randomized clinical trials (RCTs) are necessary to characterize the impact of these agents on the QT interval during anesthesia definitively [10, 14].

Given the gaps in current knowledge, this study aims to provide a comparative analysis of the QT interval changes induced by Atracurium and Cisatracurium during general anesthesia. By investigating these effects in a controlled setting, this study offers anesthesiologists critical insights into the cardiac safety profiles of these two neuromuscular blocking agents (NMBAs), ultimately guiding safer perioperative care for patients at risk of arrhythmia.

## Method

### Trial design and settings

This study was a parallel-group double-blind randomized controlled trial (RCT) conducted at *Farshchian (Sina) Hospital* in Hamedan, Iran, in 2022–2023. The RCT adhered to the CONSORT guidelines and aimed to evaluate and compare the effects of two muscle relaxants, Atracurium and Cisatracurium, on the QTc interval in patients undergoing cataract surgery. The study was registered with the Iranian Registry of Clinical Trials (IRCT) under the registration number IRCT20120215009014N441, with registration on September 10, 2022.

### Subject selection

The study sample included 80 patients over the age of 40 who were scheduled for cataract surgery under general anesthesia at *Farshchian (Sina) Hospital*. Using the random block method, the participants were randomly assigned in a 1:1 ratio to two experimental groups: one receiving Atracurium and the other receiving Cisatracurium. Both researchers and participants were blinded to group assignments. Patients were given a four-digit code that remained confidential until the data analysis was completed. Only one member of the research team held the key to the codes.

### Sample size calculation

The sample size was calculated using the test for the difference in means between two independent samples (Formula 1). The necessary values for the sample size formula were derived from a similar study by Mellinghoff et al. [16], with a test power of 90% and a type I error rate of 5%. A total of 40 samples were required per group, leading to a combined sample size of 80.

$$n = \frac{{{{({z_{1 - \alpha /2}} + {z_{1 - \beta }})}^2} \times [{\delta _1}^2 + {\delta _2}^2]}}{{{{({\mu _1} - {\mu _2})}^2}}}$$

$$\:n=\frac{{(1.96+1.282)}^{2}\times\:[{1.00}^{2}+{1.1}^{2}]}{{(3.1-2.3)}^{2}}=36.29\sim40$$

### Randomization procedures and blinding procedures

In this randomized clinical trial (RCT), a randomized block design was implemented, utilizing a computer-generated random number sequence to allocate participants to either the Atracurium or Cisatracurium group. This approach facilitated balanced group assignment and reduced selection bias by ensuring an equal number of participants in each group throughout the study. Additionally, a double-blind method was employed, meaning that neither the participants nor the anesthesiology team knew which muscle relaxant was given until the trial concluded.

### Eligibility criteria

Participants had to have had full consciousness before surgery, with no conditions that could impair this state, such as dementia. They did not have a history of known conduction disorders, including long QT syndrome, or allergies to the anesthetic agents used in the study. Additionally, participants were required not to have taken any medications that could affect the QT interval, such as antiarrhythmics, antidepressants, antipsychotics, or chemotherapy agents. Stable electrolyte levels were necessary, and participants had to be non-alcoholic, non-smokers, and over 40 years of age.

Participants were excluded if they required invasive procedures, such as tracheal intubation, before the electrocardiogram, or if there was a need to renew or increase the dosage of muscle relaxants or other medications that could impact the QT interval. Other exclusion factors included electrolyte imbalances, liver disease, a history of alcoholism or smoking, thyroid disorders (specifically hyperthyroidism), and a prior history of cardiac surgery.

### Participant preparation and anesthesia protocol

A researcher-designed checklist was employed to record demographic information, medical history, and examinations before, during, and after the interventions. Before anesthesia induction, patients’ vital signs, including respiratory rate, blood oxygen saturation, blood pressure, heart rate, and body temperature, were documented in the checklist. Additionally, a long lead (V2) electrocardiogram (ECG) was obtained under standard conditions, with the time, date, research code, and patient’s name recorded and filed in their research record.

All participants received standard anesthesia induction, which consisted of Fentanyl (1–2 µg/kg/dose intranasally), Lidocaine (1 mg/kg), and Propofol (2 mg/kg), accompanied by a muscle relaxant. One group received Atracurium at a dose of 0.25 mg/kg, while the other group received Cisatracurium at a dose of 0.05 mg/kg. Atracurium (ATC code M03AC04) was manufactured by *Caspian Company*, and Cisatracurium (ATC code M03AC11) was produced by *Alborz Darou Company*. Both muscle relaxants were administered simultaneously as a bolus intravenous dose with the induction of anesthesia.

### Monitoring and assessment during anesthesia

After induction, a laryngeal mask airway was placed for all patients. Mechanical ventilation settings were adjusted according to the patient’s needs, including tidal volume, respiratory rate, and inspiratory oxygen concentration, to ensure adequate ventilation throughout the procedure. Anesthesia maintenance was achieved with a continuous infusion of Propofol and the use of inhalational agents, if required, while monitoring the depth of anesthesia to ensure optimal conditions during surgery.

The administration and effects of muscle relaxants were monitored throughout the procedure using a train-of-four (TOF) stimulation method, assessing the degree of neuromuscular blockade. Patients’ conditions, including vital signs and any adverse effects, were continuously monitored until the end of recovery. The ECGs were printed at several time points: baseline (before anesthesia) (T0), five minutes post-anesthesia induction (T1), after the surgery (T2), and after recovery (T3). The QT interval was measured from the beginning of the QRS complex to the end of the T wave and corrected to obtain the QTc using Bazett’s formula [17]. The electrocardiograph device used was a three-channel model manufactured by *Nihon Kohden*, and its accuracy functionality was verified according to the hospital’s medical equipment guidelines.

### Ethical considerations

This study was performed by the ethical standards in the 1964 Declaration of Helsinki and its later amendments or comparable ethical standards. The research was approved by the University’s Research Council and carried out after obtaining ethical approval from the Ethics Committee of Hamadan University of Medical Sciences (UMSHA), Iran (Ethics code: IR.UMSHA.REC.1400.843, Ethics approval date: 2022-01-22). All participants were provided with a written informed consent form detailing the conditions of participation in the study, as well as potential benefits and risks. Participants were assured that their participation, non-participation, or withdrawal from the study would not affect their standard diagnostic and treatment procedures. They were also assured that if any adverse effects from the experimental interventions occurred, necessary actions would be taken immediately and without any cost to the participant. The researchers ensured that all participants had valid health insurance. No additional costs were imposed on the participants for diagnostic or therapeutic procedures outside the standard treatment protocol, such as a second electrocardiogram. Finally, participants were assured that their personal information would remain confidential with the research team, and the results of the study would be published in aggregate form without any identifiable information.

### Data analysis

The data distribution was initially assessed using the Kolmogorov-Smirnov test to determine normality. Since the data followed a normal distribution, the chi-square test was employed to compare the frequency of qualitative variables between the groups. For the comparison of quantitative data, the independent t-test was utilized to compare the means across different groups. Furthermore, the paired t-test was used to evaluate the changes in the mean QTc interval at various time points within each experimental group. In addition to these tests, descriptive statistics were used to summarize the participants’ demographic data and other baseline characteristics. Continuous variables were expressed as mean ± standard deviation (± SD), while categorical variables were presented as frequencies and percentages. A *p*-value of less than 0.05 was considered statistically significant. All statistical analyses were performed using SPSS version 29, ensuring that the assumptions of each statistical test were met.

## Results

### Demographic characteristics of the patients

**Fig. 1:**
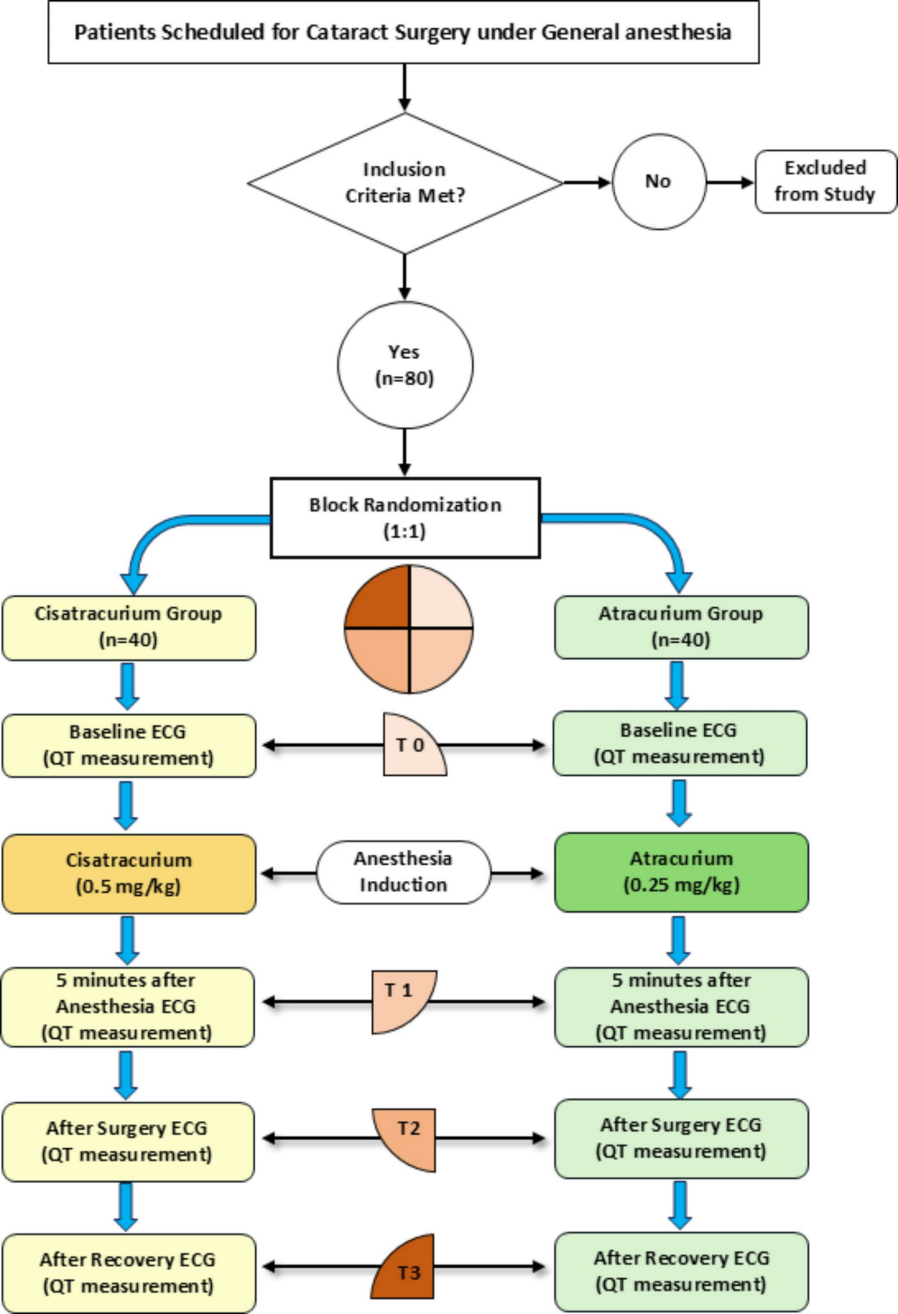
Schematic representation of the parallel-group double-blind RCT design

Table 1 represents the demographic characteristics of the patients, and Fig. 1 demonstrates a summary of the flowchart of patient allocation and analysis.

In this RCT, a total of 80 patients with cataracts were included and divided into two experimental groups: Cisatracurium (*n* = 40) and Atracurium (*n* = 40). The study population consisted of 41 males (22 in the Cisatracurium group and 19 in the Atracurium group) and 39 females (18 in the Cisatracurium group and 21 in the Atracurium group), with no significant difference in gender distribution between the two groups (*P* = 0.502) (Fig. 1).

Regarding underlying diseases, 64 patients had a history of underlying conditions (31 in the Cisatracurium group and 33 in the Atracurium group), while 16 patients did not have any such history (9 in the Cisatracurium group and 7 in the Atracurium group). The distribution of patients with and without underlying diseases was comparable between the two groups (*P* = 0.576 for those with a history and *P* = 0.261 for those without).

The mean age of patients in the Cisatracurium and Atracurium groups did not differ significantly (*P* = 0.182). Additionally, vital parameters, including heart rate (HR), respiratory rate (RR), systolic blood pressure (BP), and diastolic BP, showed no significant differences between the two groups (Table 1).

**Table 1:** Basic characteristics of the patients

Variable		Cisatracurium (*n* = 40)	Atracurium (*n* = 40)	*P*-value
Gender	Male (*n* = 41)	22 (53.66%)	19 (46.34%)	0.502
	Female (*n* = 39)	18 (46.15%)	21 (53.85%)	0.502
History of CVDs	Yes (*n* = 64)	31 (48.44%)	33 (51.56%)	0.576
	No (*n* = 16)	9 (56.25%)	7 (43.75%)	0.261
Age	(years)	68.07 (± 12.13)	65.72 (± 10.87)	0.182
Heart rate (HR)	(b.p.m)	77.40 (± 11.70)	77.87 (± 13.98)	0.434
Respiratory rate (RR)	(r.b.p)	16.10 (± 1.80)	15.45 (± 2.42)	0.089
Systolic BP	mm.Hg	139.9 (± 15.4)	144.45 (± 19.89)	0.261
Diastolic BP	mm.Hg	89.0 (± 9.06)	87.5 (± 12.32)	0.268
Oxygen saturation	(%)	96.52 (± 2.84)	95.27 (± 3.38)	0.039*

(Mean (± SD), *: *P* value < 0.05; BP: blood pressure; b.p.m: beats per minute; r.p.m: respiration per minute; mm.Hg: millimeters of mercury)

### Comparison of QTc in the cisatracurium group

In the Cisatracurium group, a significant reduction in the QTc interval was also noted, particularly from 5 min before anesthesia versus 5 min after anesthesia (T0-T 1: -9.325 ± 5.3785 ms, *P* = 0.045), from 5 min before anesthesia versus the end of surgery (T0-T2: -9.925 ± 5.457 ms, *P* = 0.038), and from 5 min before anesthesia versus after recovery (T0-T3: -19.359 ± 8.738 ms, *P* = 0.016). In addition, a significant increase in the QTc interval was observed between 5 min after anesthesia versus the end of surgery (T1-T2: 15.794 ± 2.497 ms, *P* = 0.002). The interval from 5 min after anesthesia versus after recovery showed a non-significant increase (T1-T3: -10.025 ± 7.662 ms, *P* = 0.099), while the increase from the end of surgery versus after recovery was significant (T2-T3: -9.425 ± 6.906 ms, *P* = 0.90). These results suggest that Cisatracurium significantly influences QTc intervals during surgery, with some changes persisting into the recovery period. (see Table 2).

### Comparison of QTc in the Atracurium group

In the Atracurium group, significant reductions in the QTc interval were also noted, particularly from 5 min before anesthesia versus 5 min after anesthesia (T0-T1: -12.825 ± 6.580 ms, *P* = 0.029) and from 5 min before anesthesia versus the end of surgery (T0-T2: -13.375 ± 5.350 ms, *P* = 0.008), and from 5 min before anesthesia versus after recovery (T0-T3: 7.426 ± 5.050 ms, *P* = 0.250). Additionally, a significant increase in the QTc interval was observed between 5 min after anesthesia versus the end of surgery (T1-T2: 32.322 ± 5.110 ms, *P* = 0.0019). The interval from 5 min after anesthesia versus after recovery showed a non-significant increase (T1-T3: 7.775 ± 5.800 ms, *P* = 0.093), while the increase from the end of surgery versus after recovery was significant (T2-T3: 8.325 ± 4.129 ms, *P* = 0.025). These results suggest that Atracurium significantly influences QTc intervals during surgery, with some changes persisting into the recovery period. (see Table 2).

**Table 2:** Comparison of mean QTc changes in the Cisatracurium and Atracurium groups at different time points (T0-T3)

Time points	Cisatracurium group (*n* = 40)		Atracurium group (*n* = 40)	
	QTc change	*P*-value	QTc change	*P*-value
5 min before anesthesia (T0) vs. 5 min after anesthesia (T1)	-9.325 (± 5.378)	0.045 *	-12.825 (± 6.580)	0.029 *
5 min before anesthesia (T0) vs. end of surgery (T2)	-9.925 (± 5.457)	0.038 *	13.375 (± 5.350)	0.008 *
5 min before anesthesia (T0) vs. after recovery (T3)	19.359 (± 8.738)	0.016 *	7.426 (± 5.050)	0.250
5 min after anesthesia (T1) vs. end of surgery (T2)	15.794 (± 2.497)	0.002 *	32.322 (± 5.110)	0.0019 *
5 min after anesthesia (T1) vs. after recovery (T2)	-10.025 (± 7.662)	0.099	7.775 (± 5.800)	0.093
end of surgery (T2) vs. after recovery (T3)	-9.425 (± 6.906)	0.90	8.325 (± 4.129)	0.025 *

(Mean (± SD); *: *P* value < 0.05; QTc: Corrected QT Interval)

### Comparison of QTc in experimental groups

**Fig. 2:**
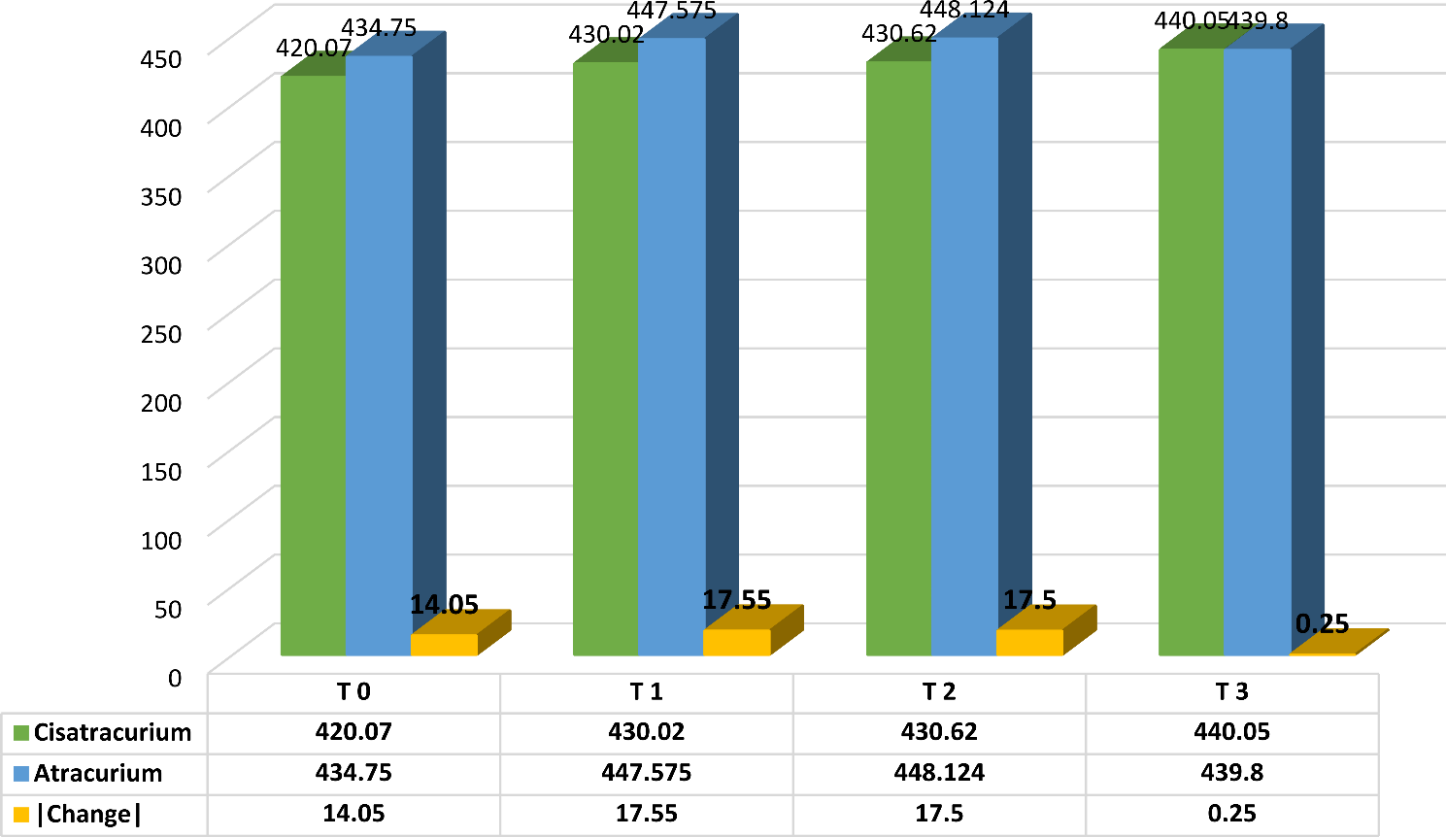
Comparison of QTc interval in two Cisatracurium and Atracurium groups in four periods. (T0: 5 min before anesthesia; T1: 5 min after anesthesia; T2: end of surgery; T3: after recovery)

Five minutes before anesthesia, the QTc interval was shorter in the Cisatracurium group compared to the Atracurium group (T0: 420.07 ± 32.30 ms vs. 434.75 ± 33.43; changes: -14.05, *P* = 0.03). Five minutes after anesthesia, the Cisatracurium group consistently exhibited shorter QTc intervals (T1: 430.02 ± 23.51 ms vs. 447.575 ± 33.43 ms; changes: -17.55, *P* = 0.004) compared to the Atracurium group. This trend continued at the end of surgery in experimental groups (T2: 430.62 ± 25.63 ms vs. 448.124 ± 33.43 ms; changes: -17.55, *P* = 0.002), with statistically significant differences. However, after recovery, the difference in QTc intervals between the two groups was no longer significant, with the Cisatracurium group showing a QTc of 440.05 ± 44.30 ms and the Atracurium group showing a QTc of 439.80 ± 33.43 ms (*P* = 0.489). These results suggest that while there are significant differences in QTc intervals between the two medicines during the surgical procedure, these differences diminish after recovery, indicating a temporary effect of the muscle relaxants on cardiac repolarization (see Table 3; Fig. 2).

**Table 3:** Comparison of QTc interval between two Cisatracurium versus Atracurium groups at various time points

	Perioperative Times	Cisatracurium (*n* = 40)	Atracurium (*n* = 40)	Change	*P*-value
T0	5 min before Anesthesia	420.07 (± 32.30)	434.75 (± 33.43)	-14.05	0.03*
T1	5 min after Anesthesia	430.02 (± 23.51)	447.575 (± 33.43)	-17.55	0.004*
T2	End of Surgery	430.62 (± 25.63)	448.124 (± 33.43)	-17.5	0.002*
T3	After Recovery	440.05 (± 44.30)	439.80 (± 33.43)	0.25	0.489

(Mean (± SD); QTc: Corrected QT Interval; *: *P* value < 0.05)

## Discussion

This clinical trial was conducted with the aim of evaluating and comparing the effects of Atracurium versus Cisatracurium as muscle relaxants used in general anesthesia, Atracurium, and Cisatracurium on the QTc interval in patients undergoing cataract surgery. The RCT involved 80 patients in two groups, one receiving Atracurium (40 patients) and the other receiving Cisatracurium (40 patients). The results indicated a significant difference in the changes in the QTc interval among patients at various time points during the surgery. However, this difference did not indicate prolonging the QTc interval to a pathological range.

The QT interval on an electrocardiogram (ECG) represents the time it takes for the heart’s electrical system to recharge between beats. Prolongation of the QT interval can lead to dangerous arrhythmias [18].

### Cisatracurium and Atracurium

The findings from both the Cisatracurium and Atracurium groups reveal that these muscle relaxants significantly influence QTc intervals during the perioperative period, with varying patterns of change. In the Cisatracurium group, a significant reduction in QTc interval was noted from 5 min before anesthesia to several key points, including 5 min after anesthesia, the end of surgery, and most notably after recovery. However, a significant increase was observed between 5 min after anesthesia and the end of surgery, highlighting the drug’s dynamic effect on cardiac repolarization. Similarly, Atracurium also led to significant reductions in the QTc interval from 5 min before anesthesia to 5 min after anesthesia and the end of surgery, while it significantly increased between 5 min after anesthesia and the end of surgery.

Similarly, Atracurium also resulted in significant reductions in QTc interval from 5 min before anesthesia to 5 min after anesthesia and the end of surgery, with a notable increase between 5 min after anesthesia and the end of surgery.

Although the changes during recovery were not consistently significant, both drugs appear to influence cardiac repolarization throughout surgery and into the recovery phase. These findings suggest that Cisatracurium and Atracurium may have differential effects on cardiac repolarization, which could have important clinical implications, particularly for patients at risk for QT prolongation.

Despite randomization and similar demographics, the observed difference in the QT interval in the Atracurium group before drug injection likely reflects normal biological and statistical variation, amplified by the small sample size. This minor variation, common in smaller studies, is within normal ranges and does not indicate a true clinical difference but a typical fluctuation that can occur in well-controlled trials with limited participants.

A few studies have compared the effectiveness of Atracurium and Cisatracurium in terms of treatment outcomes, such as in patients experiencing respiratory distress or undergoing surgery [19]. For instance, a study by Moore and colleagues [19] found no difference in treatment outcomes between the use of these medicines in managing respiratory distress patients. Additionally, a study by El-Kasaby et al. [20] demonstrated differences in the intensity and duration of the effects of these medicines at different doses during surgery. However, in our review, we did not find any studies that compared the side effects of these medicines, including cardiac or other adverse effects.

### Comparison of QTc in experimental groups

We also found that Cisatracurium prolongs the QTc interval less than Atracurium after anesthesia and at the end of surgery. This suggests that Cisatracurium may have a less pronounced effect on cardiac repolarization during the perioperative period. However, the absence of significant differences in QTc intervals after recovery indicates that the effects of these muscle relaxants on cardiac repolarization are transient. This finding is clinically relevant as it implies that while Cisatracurium might be preferable during surgery to minimize potential cardiac risks, both drugs appear to have similar safety profiles post-recovery.

Muhammad Ali et al. [21], in their RCT, compared Atracurium and Cisatracurium in patients undergoing CABG surgery. They discovered that Cisatracurium is more suited for keeping hemodynamics constant and minimizing pressure fluctuations in cardiac patients having on-pump bypass surgery.

Xuan et al. [6] investigated QTc interval changes during laryngeal mask airway insertion with and without cisatracurium. They observed that the QTc interval increased significantly after the administration of Propofol and Fentanyl but decreased after Cisatracurium injection [6]. This suggests that Cisatracurium may not significantly prolong the QTc interval during anesthetic induction.

In an RCT, Michaloudis et al. [22] showed that administration of Atracurium following midazolam did not significantly change the QTc interval. Significant QTc prolongation was observed after tracheal intubation, but the mean QTc values remained within normal limits [22].

Cisatracurium administration initially increased the QTc interval after arrival in the operating room and after Propofol and Fentanyl injection, but the QTc interval decreased after Cisatracurium injection. The QTc interval did not significantly change due to laryngeal mask airway insertion, regardless of Cisatracurium administration [6].

Anxiety may produce prolonged QTc intervals during the transfer from the ward to the surgery room. Indeed, relationships between hemodynamics, electrolytes, sympathetic and parasympathetic activity, hormone levels, temperature, and structural cardiovascular disease have all been shown to alter QTc interval length [23–25].

### Impact of Cisatracurium and Atracurium on cardiac function and ECG

Cisatracurium has proven to be particularly advantageous for patients with reduced left ventricular function, especially those undergoing open-heart surgery, due to its favorable impact on hemodynamic parameters [22, 26].

Previous studies showed different effects of Atracurium and Cisatracurium on histamine release [25, 27, 28]. Both Atracurium and Cisatracurium offer rapid onset and similar duration of action, maintaining stable hemodynamic status without triggering histamine release [29]. Cisatracurium produces NMB in the same way as Atracurium does, but without the side effects of high dosage histamine release and laudanosine buildup in the plasma [25].

OzA et al. concluded in a recent RCT that Cisatracurium acts longer than Atracurium and releases less histamine. Considering that it provides superior hemodynamic, neuromuscular, and safety profiles under comparable intubating circumstances, Cisatracurium may be a preferable substitute for Atracurium [27]. However, Cisatracurium stands out for its minimal effects on cardiovascular parameters across diverse patient populations, including healthy adults, the elderly, children, and those with severe cardiovascular disease [30].

In a RCT, Priyanka Harle et al. demonstrated that Cisatracurium at a dose of 0.15 mg/kg provides superior intubating conditions and a more stable hemodynamic profile compared to Atracurium at 0.5 mg/kg, with no signs of histamine release [31]. Additionally, Naseer et al. found that Cisatracurium leads to more stable hemodynamic indexes in patients with low ejection fraction undergoing coronary artery bypass grafting [32]. Given these findings, Cisatracurium’s consistent and favorable effects on cardiac function, particularly in vulnerable patient populations, make it a preferred choice over Atracurium in clinical settings where cardiac stability is a priority.

### Demographic variables

In this study, the demographic and baseline characteristics between the two groups were well-balanced, with no statistically significant differences in vital signs. Notably, although the oxygen saturation in the Atracurium and Cisatracurium groups was statistically significant before drug administration, this significance does not have clinical or physiological justification for affecting the QTc interval. This is because the average oxygen saturation in both groups was above 95%, which is within the normal physiological range for oxygenation in healthy individuals. Oxygen saturation levels in this range are generally considered sufficient for maintaining normal cardiac function and would not be expected to affect the QTc interval. Minor variations in oxygen saturation above 95% are not clinically significant and do not typically affect the heart’s electrical activity, including the QTc interval. Additionally, it is shown that QTc prolongation is more likely in conditions of severe hypoxia (oxygen saturation well below 90%) [33], which was not observed in either group.

Notably, we observed that blood pressure in both groups exceeded the normal limit of 135 mm Hg, likely due to preoperative stress, the non-standard conditions of measuring blood pressure in a lying position, and the advanced average age of the patients. However, since there was no significant difference between the two groups, this factor does not impact the overall results of the study.

Ultimately, this study reveals that although both Atracurium and Cisatracurium are generally safe in terms of cardiac side effects, the slight increase in the QTc interval observed with both medicines after anesthesia, as well as the significant difference seen with Atracurium compared to Cisatracurium, This homogeneity suggests that the observed differences in QTc intervals can be attributed to the pharmacological effects of the two muscle relaxants rather than differences in baseline clinical characteristics. It underscores the necessity for careful monitoring of patients before, during, and after anesthesia. Additionally, these findings highlight the need for further and more detailed studies to understand these effects better. In similar future studies, a larger sample size or separating groups based on gender could be considered to investigate further the impact of gender on the effects of medicines.

### Limitation and strength, and future suggestion

In this study, we did not face notable limitations. Various potential confounding factors, such as age, gender, body temperature, and anxiety, could influence the QT interval. However, efforts were made to standardize conditions and randomize groups, and their effects remain a concern. However, the study’s strengths include its controlled environment and randomization, which enhance the reliability of the findings and provide valuable insights into the specific anesthetic agents’ effects on the QT interval, contributing to improved clinical practices in anesthesia management. Future studies with larger sample sizes and gender-stratified analyses are recommended to elucidate further the impact of these medicines on QTc intervals and their potential implications for patient safety.

## Conclusion

In conclusion, this study showed that Atracurium causes QT prolongation compared to Cisatracurium. While both Atracurium and Cisatracurium affect QTc intervals during surgery, but Cisatracurium has a more stable and predictable effect on cardiac repolarization, making it a safer choice for patients at risk of QT prolongation. Both muscle relaxants are generally safe, but Cisatracurium’s minimal impact on cardiovascular function across diverse patient populations, including those with low ejection fraction, makes it preferred in clinical settings requiring cardiac stability.

## Data Availability

The datasets used and/or analyzed during the current study are available from the corresponding author upon reasonable request.

## Declarations

### Registration number

The research was registered with the Iranian Registry of Clinical Trials on September 10, 2022, and an IRCT code (IRCT20120215009014N441) was obtained.

## Abbreviations

QTc: Corrected QT interval
ECG: Electrocardiograms
RCT: Randomized clinical trial
